# Colorless Iodine

**Published:** 1885-04

**Authors:** 


### COLORLESS IODINE.

Under the name of *colorless tincture of iodine*, several preparations are used, depending for their popularity on the fact that they do not stain the skin. They are prepared either by the use of ammonia or of sodium sulphite or hyposulphite. They owe their particular property, or rather absence of property, to the neutralization of the iodine,and just to the extent that the iodine is decolorized is it to the same extent deprived of virtue. The free active affinity of the iodine, to which its local action must be due, is destroyed in these preparations, and the destruction is not slow or uncertain, but in two of the methods mentioned it is sufficiently rapid and definite to to be made the basis of a method of quantitative analysis. It is certainly difficult to see how any person could go so wide of simple chemical principles as to invent or employ this mixture.

*Potassium chlorate*, or, as it is still erroneously called by many, chlorate potash, is a remedy concerning which extraordinary claims have been made, based upon most erroneous notions of its chemical qualities. It is employed in the laboratory as a source of oxygen. Knowledge of this fact has led to its employment as an oxidizing agent in diseases which have been supposed to express deficient oxidation. I have nothing to say here as to the clinical results obtained from potassium chlorate in any disease—althoguh I believe it is much less in favor than formerly—but I enter a protest against any advocacy of its usefulness as an oxidizing agent. Under temperatures and conditions such as those which it meets in the human system it is one of the most stable of bodies, does not part with its oxygen or chlorate, and, indeed, will not begin to do so except under very high heat. I have found by actual experiment that ten grains of the salt kept for two hours at a temperature of 100° F. in contact with an artificial gastric juice did not develop oxidizing qualities sufficient to oxidize one sixtyeth of a grain of Phosphorus. This experiment is merely confirmatory of what everyday experience with the substance teaches.

